# Biomic Specialization and Speciation Rates in Ruminants (Cetartiodactyla, Mammalia): A Test of the Resource-Use Hypothesis at the Global Scale

**DOI:** 10.1371/journal.pone.0028749

**Published:** 2011-12-12

**Authors:** Juan L. Cantalapiedra, Manuel Hernández Fernández, Jorge Morales

**Affiliations:** 1 Departamento de Paleobiología, Museo Nacional de Ciencias Naturales (CSIC), Madrid, Spain; 2 Departamento de Paleontología, Facultad de Ciencias Geológicas, Universidad Complutense de Madrid, Madrid, Spain; 3 Departamento de Cambio Ambiental, Instituto de Geociencias (UCM-CSIC), Madrid, Spain; Instituto de Higiene e Medicina Tropical, Portugal

## Abstract

The resource-use hypothesis proposed by E.S. Vrba predicts that specialist species have higher speciation and extinction rates than generalists because they are more susceptible to environmental changes and vicariance. In this work, we test some of the predictions derived from this hypothesis on the 197 extant and recently extinct species of Ruminantia (Cetartiodactyla, Mammalia) using the biomic specialization index (BSI) of each species, which is based on its distribution within different biomes. We ran 10000 Monte Carlo simulations of our data in order to get a null distribution of BSI values against which to contrast the observed data. Additionally, we drew on a supertree of the ruminants and a phylogenetic likelihood-based method (QuaSSE) for testing whether the degree of biomic specialization affects speciation rates in ruminant lineages. Our results are consistent with the predictions of the resource-use hypothesis, which foretells a higher speciation rate of lineages restricted to a single biome (BSI = 1) and higher frequency of specialist species in biomes that underwent high degree of contraction and fragmentation during climatic cycles. Bovids and deer present differential specialization across biomes; cervids show higher specialization in biomes with a marked hydric seasonality (tropical deciduous woodlands and schlerophyllous woodlands), while bovids present higher specialization in a greater variety of biomes. This might be the result of divergent physiological constraints as well as a different biogeographic and evolutionary history.

## Introduction

Species biogeography is influenced by present environmental conditions, but it is also true that large-scale processes in the past have had a major impact on the distribution of the living forms that we see today [1]–[7]. The changing connections among landmasses, the vicariance due to the creation and alternate expansion-contraction of biomes as well as the establishment of geographic barriers have influenced the way lineages have evolved during millions of years. Some researchers have identified such large-scale processes as major forces triggering faunal turnovers and some of the hypotheses based on these ideas were gathered together in what is called the habitat theory [1]. Under this view, physical environmental changes are the main promoters of speciation and extinction, rather than biotic interactions [1], [8]–[10]. Vrba's resource-use hypothesis, which is included as part of this theory, suggests that the degree of specialization of species has an important role on the differential evolution of clades [11]–[13]. Vrba's [12] stating of this hypothesis indicates that “clades of species, whose resources have tended to disappear during the recurrent environmental extremes that they encountered during their histories, should generally have had a high incidence of strong, directional pressures, vicariance, speciation and extinction”. Specialist species are more prone to suffer such a limitation of their resources and, thus, they are more susceptible to environmental changes, vicariance and strong directional selection. Accordingly, this hypothesis predicts higher speciation and extinction rates in specialist species. On the other hand, generalists are expected to present higher flexibility, which allows them to survive through climatic cycles and to maintain slow speciation rates through time. Here, the term “resource” encompasses a wide range of physical and biotic factors, including moisture, temperature, substrate, vegetation cover, food items and any other environmental components usable by an organism [12]–[14]. In relation to this, the resource-use hypothesis differs from others in that the character “specialist” or “generalist” in a species is related to its distribution on terrestrial biomes [12] and points out the contrast between biome specialist (stenobiomic species) and biome generalist (eurybiomic species) as paramount for the establishment of differential species turnover rates. Thereby, under the resource-use hypothesis, stenobiomic lineages inhabit a particular biome and, thus, a relatively narrow range of vegetation physiognomy, which makes them more prone to suffer vicariance due to climatic forcing and the subsequent fragmentation of that biome. Conversely, eurybiomic lineages can use resources from a wider range of biomes, which allows them to overcome climatic changes and habitat fragmentation [12]. Following Vrba's ideas, Hernandez Fernández & Vrba [14] developed the biomic specialization index (BSI), which is the number of biomes inhabited by a species. The BSI is a reliable measure of species ecological specialization and, unlike others proposed [15]–17, it makes possible intercontinental and intertaxa comparisons. It also avoids the bias for rare and less-sampled taxa [18]. Here, we consider a species to be stenobiomic if its distribution is restricted to one biome or eurybiomic if it abides in more than one [12], [14], [19].

The resource-use hypothesis was originally conceived by Vrba after the study of the fossil record of African large mammal taxa [12]. However, little information about ecological features such as diet or biomic distributions is still available for most fossil ruminants, as well as for other mammalian clades, despite their extensive fossil record (more than 300 fossil genera in the case of ruminants) [20]–[22]. In addition, including only those fossil taxa for which we have ecological information would largely bias any analytical approach. For this reason, Hernández Fernández and Vrba [14] and Moreno Bofarull et al. [19] used the modern mammalian assemblages of, respectively, Africa and South America to test Vrba's resource-use hypothesis, finding strong support for several of its subsidiary predictions: 1) given a clade, we should find more specialist species than expected by chance, due to their higher rates of speciation; 2) we should expect higher proportion of stenobiomic species in biomes that underwent a high degree of fragmentation and contraction during climatic fluctuations (e.g., Milankovitch cycles), since populations in those biomes are subject to a high incidence of vicariance; 3) different clades are expected to show different degrees of specialization, because they may be adapted to very different climates and environments.

Here, we tested these predictions using both non-phylogenetic and phylogenetic approaches. Firstly, following the method by Hernández Fernández and Vrba [14], we compared our biomic presence–absence matrix with those generated by null models: Monte Carlo randomizations of the observed data that produced a distribution of BSIs expected in the presence of a random evolutionary process where biomic specialists would be randomly distributed across biomes and clades [23]. This method allows testing hypothesis connecting ecological specialisation and macroevolutionary processes. Secondly, we tested the first prediction, which links higher speciation rates to more specialized lineages, against other evolutionary scenarios by applying a phylogenetic likelihood-based method (QuaSSE) that fits quantitative-trait-dependent models of speciation-extinction.

The use of these different analyses allows us to test the predictions stated above against other scenarios. The Monte Carlo randomizations assess if the observed biomic distribution is different from an evolutionary scenario where biomic specialization is independent from clades and biomes. By using QuaSSE, we have compared four different trait-dependent models on how the biomic specialization could affect speciation rates.

**Figure 1:**
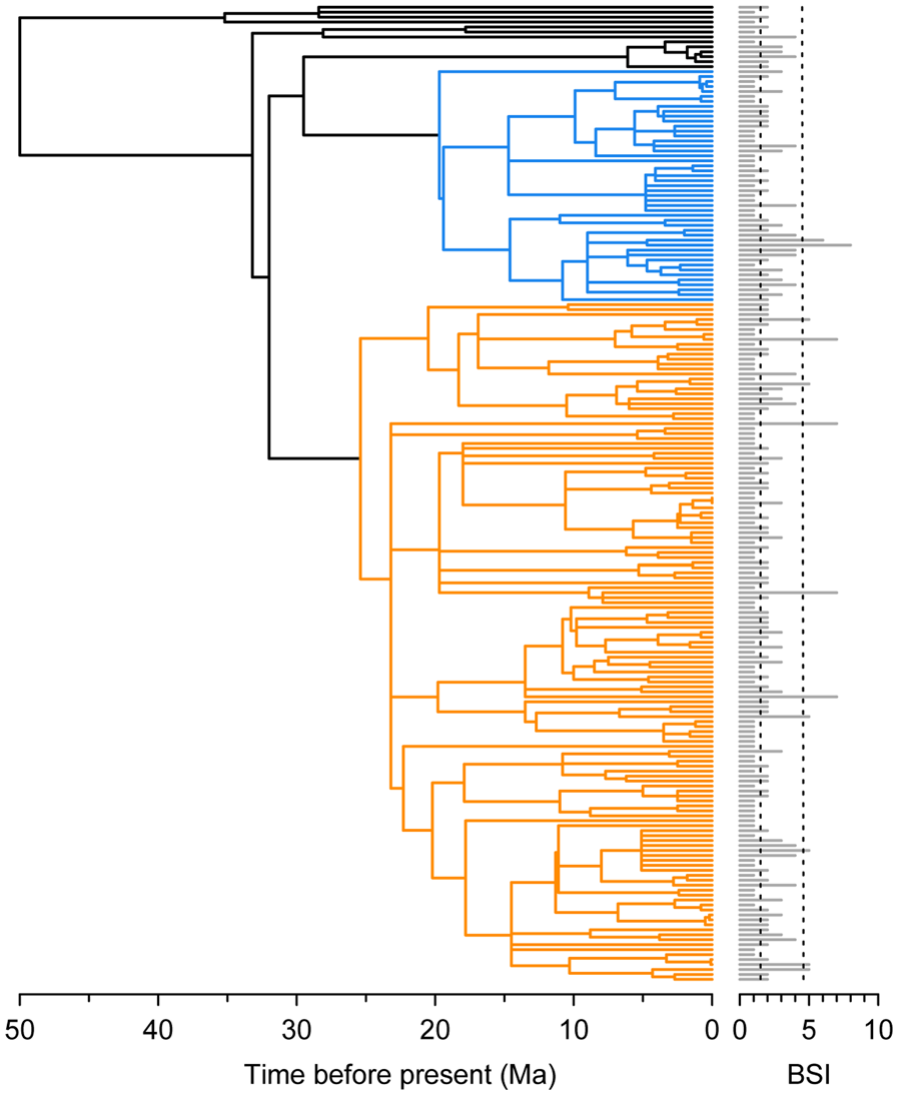
Phylogenetic tree of the ruminants [**60**] and BSI values distribution. Shown with colours are the most species rich families: Cervidae (blue) and Bovidae (orange). BSI is shown by the horizontal bar for each species. The vertical dashed lines indicate the limits between stenobiomic species (BSI = 1), semi-eurybiomic species (1<BSI<5) and extreme eurybiomic species (BSI≥5).

We conducted our analyses on the 197 extant and recently extinct species of ruminants, their BSI dataset and a phylogeny of the group (Fig. 1). Ruminants (Ruminantia Scopoli, 1777) [24] are distributed world-wide, naturally occurring in five continents, present a high diversity of ecological adaptations and inhabit in all of the world's biomes. All these particular features make ruminants a group of major interest in studies on macroevolution and ecology, and allowed us to compare, for the first time, the biomic specialization among all the biomes at the global scale.

We found that a high frequency of ruminant species is restricted to a single biome as a consequence of high speciation rates in stenobiomic lineages and higher specialization of species inhabiting biomes that underwent a high degree of fragmentation and contraction during climatic shifts. Finally, our results show a disparate specialization across biomes in bovid and cervids owing to their different biogeographic histories, resource requirements and physiological adaptations.

## Results

### Distribution of the Biomic Specialization Index (BSI)

**Figure 2:**
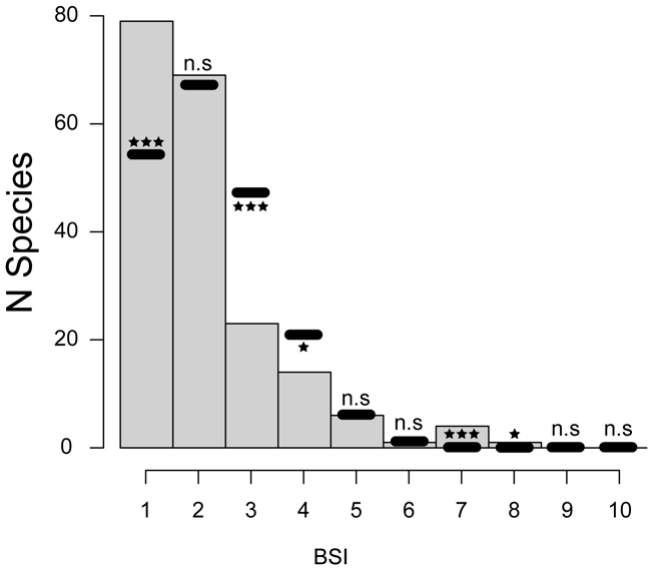
Frequency distribution of biomic specialization index (BSI) for ruminants. Grey bars represent observed distribution of BSI in Ruminantia. Black lines show the average number of species from 10000 Monte Carlo Simulations (Table 1). Significance level for the difference between the observed and the expected distributions: ***, p<0.001; **, 0.01>p>0.001; *, 0.05>p>0.01; n.s., not significant.

The frequency distribution of BSI for ruminants is powerfully right-skewed (Fig. 2). Mean BSI among ruminants is 2.10, with 79 species (40.1%) inhabiting only one biome and 69 (35.03%) inhabiting two biomes. Conversely, only the 6.10% of the species inhabits five or more different biomes (BSI≥5), with *Odocoileus* being the only genus inhabiting eight different biomes. Our results pinpoint a significantly higher proportion of biomic specialist species (BSI = 1) than expected by random draws (Table 1). The frequency of species with BSI = 2 is not significantly different than the expected by chance, while the proportion of species inhabiting three (BSI = 3) and four biomes (BSI = 4) is significantly lower than expected. We found non-significant differences in proportions for species with BSI = 5 and 6 between the observed values and those yielded by the 10000 simulations. Nevertheless, the frequency of ruminant species with BSI = 7 is significantly higher than expected by chance. Finally, although the Monte Carlo simulations yielded small percentages of species inhabiting nine and ten biomes, there is no statistically significant difference with the absence of these highly eurybiomic species in our data set (Table 1).

**Table 1:** Observed and simulated BSI values for ruminants.

BSI	%	Monte Carlo Analysis			
		Mean %	Std.dev	Range	*p*
1	40.10	27.47	2.60	18.00–38.00	<0.001
2	35.00	34.16	3.50	22.00–49.00	0.665
3	11.70	23.94	3.10	13.00–36.00	*<0.001*
4	7.10	10.63	2.10	2.80–19.00	*0.045*
5	3.05	3.11	1.20	0.00–7.90	*0.983*
6	0.51	0.61	0.56	0.00–3.40	*0.470*
7	2.03	0.08	0.20	0.00–1.70	<0.001
8	0.51	0.01	0.06	0.00–1.10	0.011
9	0.00	0.00	0.01	0.00–0.57	1.000
10	0.00	0.00	0.01	0.00–0.56	1.000

Frequencies of ruminant species in each BSI and comparison with 10000 Monte Carlo simulations. *%*, proportion of the total number of species (197); *p*, probability of species in the simulations being greater than or equal to (plain) or lower than or equal to (italics) the observed proportion in ruminants.

**Figure 3:**
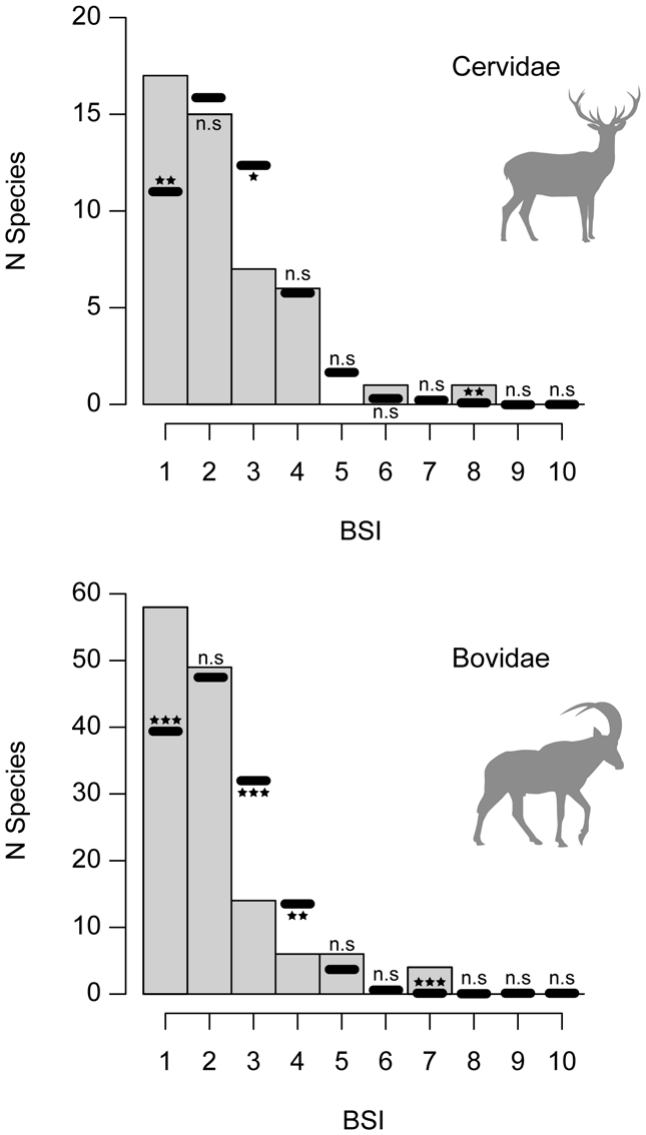
Frequency distribution of biomic specialization index (BSI) for Cervidae and Bovidae. Grey bars represent observed distribution of BSI. Black lines show the average number of species from 10000 Monte Carlo Simulations (Table 2 and 3). Significance level for the difference between the observed and the expected distributions: ***, p<0.001; **, 0.01>p>0.001; *, 0.05>p>0.01; n.s., not significant.

The comparison between the real distribution of BSI values in Cervidae and Bovidae and the distribution obtained from 10000 Monte Carlo simulations, are shown in Fig. 3 and Tables 2 and 3. Mean BSI in Cervidae is 2.25 and 2.04 in Bovidae. Both families present a right-skewed distribution of BSI and a significantly higher proportion of stenobiomic species (BSI = 1) than the expectations from random draws, which follow the observed trend of Ruminantia. Bovidae present a slightly higher percentage of specialists (42.3%) than Cervidae (36.2%). The proportion of species with BSI = 2 does not differ from what we would expect by chance, while species inhabiting three biomes are more scant than expected in both clades. Bovidae has higher proportion of extreme eurybiomic species (BSI≥5) than Cervidae (7.3% against 4.26%).

**Table 2:** Observed and simulated BSI values for Cervidae.

BSI	%	Monte Carlo Analysis			
		Mean %	Std.dev	Range	*p*
1	36.20	23.47	5.00	2.50–43.00	0.005
2	31.90	33.73	6.90	11.00–63.00	*0.709*
3	14.90	26.28	6.40	4.30–54.00	*0.031*
4	12.80	12.23	4.40	0.00–32.00	0.832
5	0.00	3.58	2.60	0.00–16.00	*0.172*
6	2.13	0.63	1.20	0.00–9.50	0.320
7	0.00	0.08	0.42	0.00–4.90	*0.968*
8	2.13	0.00	0.09	0.00–2.40	0.001
9	0.00	0.00	0.00	0.00–0.00	1.000
10	0.00	0.00	0.00	0.00–0.00	1.000

Frequencies of species within Cervidae in each BSI and comparison with 10000 Monte Carlo simulations. *%*, proportion of the total number of species (47); *p*, probability of species in the simulations being greater than or equal to (plain) or lower than or equal to (italics) the observed proportion in Cervidae.

**Table 3:** Observed and simulated BSI values for Bovidae.

BSI	%	Monte Carlo Analysis			
		Mean %	Std.dev	Range	*p*
1	42.30	28.77	3.20	16.00–40.00	<0,001
2	35.80	34.63	4.20	18.00–51.00	0.656
3	10.20	23.39	3.60	10.00–38.00	*<0,001*
4	4.38	9.88	2.40	1.50–20.00	*0.007*
5	4.38	2.76	1.40	0.00–10.00	0.128
6	0.00	0.51	0.62	0.00–4.10	*0.520*
7	2.92	0.06	0.22	0.00–1.70	<0,001
8	0.00	0.00	0.06	0.00–0.86	*0.994*
9	0.00	0.00	0.01	0.00–0.83	1.000
10	0.00	0.00	0.00	0.00–0.00	1.000

Frequencies of species within Bovidae in each BSI and comparison with 10000 Monte Carlo simulations. *%*, proportion of the total number of species (137); *p*, probability of species in the simulations being greater than or equal to (plain) or lower than or equal to (italics) the observed proportion in Bovidae.

### Effect of BSI on speciation rates

**Figure 4:**
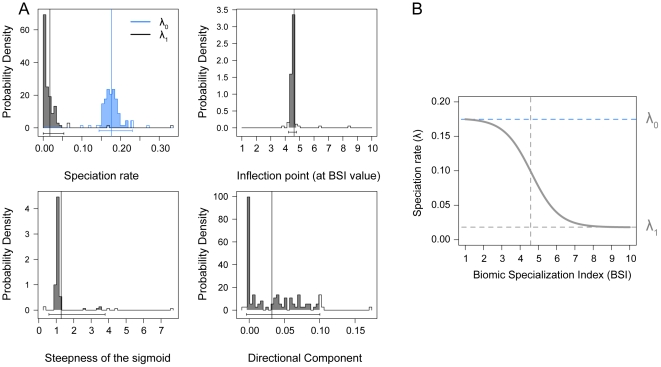
Speciation rate sigmoid model fit. Probability distributions of the parameters from the 100 trees analysed (A) and graphic representation of the sigmoid model under QuaSSE (B). λ_0_, speciation rate for low values of BSI; λ_1_, speciation rate for high values of BSI. Mean values are indicated by the solid vertical lines. Bars at the bottom of the distributions and the shaded areas correspond to the 95% credibility intervals. Under QuaSSE, the speciation rate for low BSI values are almost tenfold the speciation rate for lineages with high BSI (from 0.17 to 0.018), with an inflection point at 4.61.

Table 4 shows the mean AIC scores for the competing models. Among trait-based speciation and extinction models, there was a strong support for a model in which the speciations rates were inferred to decrease with decreasing biomic specialization (increasing BSI) following a sigmoidal function (Table 4). This model obtained the best AIC score. Sigmoid models with a small directional component of character evolution and without such deterministic term were alternatively chosen as best model along the 100 cases (ΔAIC = 0.4) and, therefore, does not allow us to determine the presence or absence of directional evolution of biomic specialization through the history of ruminants. The best model depicts a mean speciation rate of 0.17 for lineages with BSI = 1 that starts to decrease immediately reaching a inflection point at BSI = 4.6 and dropping to speciation rates of around 0.018 in extreme eurybiomic lineages (Fig. 4).

**Table 4:** Fits of the quantitative-trait-dependent speciation models (QuaSSE).

Model Type	n	lnL	lnL Range	St.Dev	AIC	AIC Range	St.Dev	ΔAIC
Constant λ	3	−1085.5	−1332.1–−1076.8	43.8	2241.5	2159.5–2670.2	87.6	34.3
Linear λ	4	−1081.7	−1327.5–−1073.4	43.2	2235.3	2154.8–2662.9	86.3	28.1
Sigmoidal λ	6	−1076.6	−1149.4–−1067.3	18.1	2207.7	2146.6–2310.8	36.1	0.4
Modal λ	6	−1084.5	−1158.8–−1047.1	18.8	2217.5	2106.3–2329.5	37.7	10.2
*Directional Tendency*								
Linear λ	5	−1079.3	−1325.6–−1073.1	39.0	2226.4	2156.3–2661.1	78.0	19.1
**Sigmoidal λ**	**7**	**−1073.1**	**−1144.4**–**−1066.6**	**17.8**	**2207.3**	**2147.2–2302.9**	**35.5**	**0.0**
Modal λ	7	−1075.6	−1158.8–−1046.5	19.2	2213.6	2106.9–2331.5	38.4	6.3

Summary of model fits for the correlation between biomic specialization index (BSI) and speciation rates for ruminants. *lnL*, log Likelihood of the fit; *n* number of parameters; *AIC*, Akaike Information Criterion; *ΔAIC*, difference of *AIC* relative to the best model (sigmoidal λ with directional evolution, in bold). Mean and range of *lnL*, *AIC* and *ΔAIC* from the 100 trees analysed are shown. *St.Dev*, standard deviation.

### Proportion of biomic specialists in each biome

There are five biomes with higher proportions of specialist species than expected by chance (Table 5). The tropical rainforest (I) houses 44 ruminant species, of which around a 27% are restricted to it. 93 species inhabit the tropical deciduous woodland (II), a biome that present more than 30% of specialist species (29 spp.). The sub-tropical deserts (III) harbour 31.43% of biome specialists, being the biome with higher degree of specialization. We also found more specialists than expected in the sclerophyllous woodland (IV; 23%). The steppes and cold-deserts (VII) represent the only temperate biome housing a significantly high degree of stenobiomic species (20%). On the contrary, we also found that the taiga (VIII) harbours significantly less specialist ruminant species that expected from our Monte Carlo simulations, only a 2.5%.

**Table 5:** Observed and simulated distribution of stenobiomic ruminant species (BSI = 1) across biomes.

Biome	Ruminantia			Monte Carlo Analysis			
	sp	sp (BSI = 1)	%	Mean %	Std.dev.	Range	*p*
I	44	12	27.27	11.00	4.54	0.00–31.82	<0.001
II	93	29	31.18	16.20	3.42	3.23–29.03	<0.001
II/III	48	5	10.42	11.40	4.40	0.00–29.17	*0.907*
III	35	11	31.43	10.50	5.05	0.00–31.43	<0.001
IV	26	6	23.08	9.84	5.63	0.00–34.62	0.007
V	44	3	6.82	11.00	4.57	0.00–34.09	*0.129*
VI	29	2	6.90	10.00	5.42	0.00–34.48	*0.236*
VII	45	9	20.00	11.10	4.49	0.00–31.11	0.019
VIII	40	1	2.50	10.70	4.71	0.00–30.00	*0.008*
IX	10	1	10.00	8.93	9.03	0.00–60.00	0.285

sp., number of species; *%*, proportion of species with BSI = 1 in relation to total number of species in each biome; *p*, probability in each biome of the proportion of species with BSI = 1 being greater than or equal to (plain) or lower than or equal to (italics) the observed proportion in ruminants.

A significantly high percentage of specialist cervids was found in two biomes (Table 6): tropical deciduous woodland (II) and sclerophyllous woodland (IV). On the other hand, more than 30% of the bovids species dwelling in the tropical rain forests (I), the tropical deciduous woodlands (II) and the sub-tropical deserts (III) are biomic specialists (Table 7), and 20% of the bovids inhabiting the steppes and cold-deserts (VII) are exclusive of this biome. We also found that three biomes house no specialist bovid species, which is less than expected under a null distribution (Table 7). These biomes are the temperate evergreen forest (V), the broad-leaf deciduous forest (VI) and the taiga (VIII).

**Table 6:** Observed and simulated distribution of stenobiomic deer species (BSI = 1) across biomes.

Biome	Cervidae			Monte Carlo Analysis			
	sp	sp (BSI = 1)	%	Mean %	Std.dev.	Range	*p*
I	15	1	6.67	9.26	7.09	0.00–40.00	*0.705*
II	25	8	32.00	13.40	6.09	0.00–40.00	0.001
II/III	7	0	0.00	7.45	9.80	0.00–57.14	*0.732*
III	2	0	0.00	6.46	17.30	0.00–100.00	*0.143*
IV	5	2	40.00	6.97	11.30	0.00–80.00	0.003
V	18	3	16.67	10.30	6.74	0.00–44.44	0.102
VI	11	1	9.09	8.28	8.02	0.00–45.45	0.285
VII	10	1	10.00	7.97	8.40	0.00–50.00	0.223
VIII	12	1	8.33	8.43	7.79	0.00–41.67	*0.373*
IX	1	0	0.00	6.25	24.20	0.00–100.00	*0.067*

sp., number of species; %, proportion of species with BSI = 1 in relation to total number of species in each biome; *p*, probability in each biome of the proportion of species with BSI = 1 being greater than or equal to (plain) or lower than or equal to (italics) the observed proportion in Cervidae.

**Table 7:** Observed and simulated distribution of stenobiomic bovid species (BSI = 1) across biomes.

Biome	Bovidae			Monte Carlo Analysis			
	sp	sp (BSI = 1)	%	Mean %	Std.dev.	Range	*p*
I	25	8	32.00	11.10	6.10	0.00–44.00	<0.001
II	65	21	32.31	17.40	4.20	3.08–33.85	<0.001
II/III	38	5	13.16	12.70	5.12	0.00–34.21	0.540
III	32	11	34.38	11.90	5.51	0.00–40.62	<0.001
IV	21	4	19.05	10.70	6.55	0.00–42.86	0.064
V	25	0	0.00	11.10	6.05	0.00–40.00	*<0.001*
VI	13	0	0.00	10.20	8.26	0.00–46.15	*<0.001*
VII	29	8	27.59	11.60	5.65	0.00–37.93	0.002
VIII	23	0	0.00	11.00	6.33	0.00–39.13	*<0.001*
IX	9	1	11.11	9.86	9.84	0.00–66.67	0.281

sp., number of species; %, proportion of species with BSI = 1 in relation to total number of species in each biome; *p*, probability in each biome of the proportion of species with BSI = 1 being greater than or equal to (plain) or lower than or equal to (italics) the observed proportion in Bovidae.

## Discussion

### Frequency and speciation rate of biomic specialists

Our results show a significantly higher proportion of biomic specialist species (BSI = 1) than expected by random draws (Table 1) which can be interpreted as a direct result of higher net diversification rates in stenobiomic lineages, accordingly to the QuaSSE mocdel (Fig. 4). This outcome agrees with the prediction of the resource-use hypothesis [11], [12] and is coherent with the results obtained by Hernández Fernández and Vrba [14] for the African large mammals and Moreno Bofarull et al. [19] for the entire assemblage of South American mammals. While in these previous works conclusions on speciation rates were constructed exclusively on BSI distributions, we here directly tested for higher rates of speciation in stenobiomic lineages and found support for Vrba's hypothesis. We also found that extreme eurybiomic species with BSI = 7 and 8 are significantly more common among ruminants than expected by random simulations (Tables 1, 2 and 3; see also Figures 2 and 3), as previously noticed by Hernández Fernández and Vrba [14] and Moreno Bofarull et al. [19]. Nevertheless, under QuaSSE, extreme eurybiomic lineages show low rates of speciation (around a tenth of the speciation rate in specialists; Fig. 4). Hence, it must be addressed that these super-generalists should possess also low rates of extinction as a result of their high ecological flexibility, which allows them to survive through multiple climatic cycles, as suggested by Hernández Fernández and Vrba [14]. In any case, no ruminant occupies all the ten biomes, since occupying all extreme biomes requires an unachievable degree of versatility [14].

### Specialization across biomes

The resource use hypothesis predicts higher specialization of species inhabiting biomes that underwent a high degree of fragmentation and contraction during climatic cycles [12], [1], [8]. At the global scale, these biomes are located at extreme climatic conditions: tropical rain forest (I), subtropical desert (III), steppe (VII) and tundra (IX). As yielded by our Monte Carlo analyses, ruminant biomic specialization in tropical rain forests (I), sub-tropical deserts (III) and steppes (VII) is in concert with such prediction (Table 5). Nevertheless, we also found some interesting exceptions. Our analyses revealed that the tropical deciduous woodlands (II) also present a significantly higher percentage of specialist species than expected under random modeling (31.18%). This biome has already been reported as harbouring a high proportion of specialists. For example, Hernández Fernández and Vrba [14] found that a 19.9% of the large mammals in African tropical deciduous woodlands were stenobiomic. Despite not representing a climatic extreme, they argued that this biome, which is in close association with the rainforest (I), did also undergo expansions and retractions of its area during climatic episodes, providing a suitable situation for the creation of patches and refuges where speciation and specialization took place [1]. Furthermore, ruminants are more restricted to a particular vegetation physiognomy and, thus, they are prone to be more stenobiomic than insectivorous, omnivorous or carnivorous clades [11], [25]. The tropical deciduous woodlands are characterised by a seasonal leaf fall, which represents an important decrease in the resources during extremely dry months of the year. Both Cervidae and Bovidae also constitute high percentages of specialists in this biome (Table 6 and Table 7), which supports this trend towards specialization in strongly seasonal deciduous landscapes. This is probably the cause behind the significantly higher proportion of biomic specialist in the sclerophyllous woodland (IV), which shows a 23% of species restricted to it (Table 5) and is not located at a climatic extreme either. Nevertheless, this specialization in the sclerophyllous woodland (IV) is especially marked in cervids (Table 6).

On the other hand, the tundra (IX) does not present as high percentages of ruminant specialist species as predicted by the resource-use hypothesis (Table 5). We can find a cause for this outcome in the method of codifying the BSI. The percentage of species inhabiting the tundra includes those inhabiting analogous vegetation belts in mountains (see Methods): species of goats and other ruminants that are not constrained to the high mountain landscape, but dwell also in several different altitudinal ranges depending on seasonality and the availability of food [26]–[29]. With the purpose of exploring the behaviour of the high latitude tundra dwellers we repeated the Monte Carlo simulations and compared our data after excluding all the species inhabiting mountain ranges. We obtained that 50% of the species abiding the tundra (IX) are tundra-specialists, a proportion significantly higher than expected from random modeling (p = 0.008) and consistent with the predictions of the resource-use hypothesis. Excluding the taxa abiding mountain ranges from our analyses yielded similar results in other biomes, increasing the proportion of biomic specialists that they harbour, although it does not affect the significance levels of the original analysis.

Noteworthy is the fact that there are significantly less specialist species in the taiga than expected from random draws. The taiga is probably the biome with largest geographical extent today, and its species present extensive distributions [27], [28]. In addition, only two main climatic dominions are recognized today and although some fragmentation has been reported for this biome through past climatic cycles [30], this has been relatively reduced in comparison with other biomes. These facts do not favour vicariance and speciation of its specialist species.

### Differential specialization among clades

The mean value of BSI in Cervidae (2.25) and Bovidae (2.04) is very similar to the mean BSI of Ruminantia (2.10), and the right-skewed distributions of their BSI values largely resemble that of ruminants' (Fig. 2 and 3). Thus, we can state that the degree of biomic specialization is similar among these clades in terms of BSI distributions, although they differ in their specialization across biomes (Table 6 and 7). Bovidae presents a significantly higher percentage of specialists than expected in four out from ten biomes (I, II, III and VII), whereas Cervidae only in two (II and IV). It seems that differences in biogeographic history, resource requirements and adaptations have marked the evolution of these two ruminant clades. In Africa the tropical rainforest (I) is widely distributed and is home for a high number of specialist bovids (Table 7). Afrotropical ruminant faunas are entirely dominated by bovids whereas tropical cervids, which never entered into the Afrotropics, are found in Asia and the Neotropics. In the Indomalaysian biogeographic region, the tropical rainforest is highly reduced and usually associated with mountainous ranges. In order to maintain genetically viable populations, the species in these areas usually inhabit in several ecosystems (biomes) due to altitudinal zonation. Such is the case of some species of the cervid genus *Rusa*, which are found in islands of the Indomalaysian region where their distributions range from the sea level up to 2000 m, including different vegetation belts [31]. The species of the genus *Muntiacus*, another group of Indomalaysian cervids, also show distributions that include mountainous ranges. The ruminants of the Neotropics are the result of a radiation of generalists that dispersed into South America when the Panamanian land-bridge appeared ca. 3.5 Ma [1], [32], [5]. Additionally, most of the species are also associated to mountains and only a few species of the genus *Mazama* exclusively inhabit the lowlands of the Amazonas. These few species probably have not proliferated in the Amazon Basin during climate cycles because their potential niches were already occupied by large species of South American rodents, such as pacas (Agoutidae) and agoutis (Dasyproctidae). In general we could say that tropical rainforests (I) in Africa have capacity to maintain more biomic specialist ruminants than in the Indomalaysian region and the Neotropics owing to their different geographic constraints and evolutionary history. Furthermore, the cladogenesis of bovids has been closely related to the sub-Saharan tropics. The responses of African extinct bovids to climatic fluctuations and the appearance and spread of more arid environments (savannah woodlands and savannah grasslands) during the Neogene have been well documented [33]. During the late Miocene and Pliocene, a period marked by a global increase in seasonality and the appearance of open grasslands [34], [35], a range of bovid tribes adapted to these new savannah-like environments emerged (Aepycerotini, Alcelaphini, Hippotragini, Reduncini and Tragelaphini). Subsequent radiations during the Plio-Pleistocene, coincident with massive cooling pulses, gave rise to some of the most successful bovid genera we see today [36]–[38]. Vrba's works on the African fossil record highlighted a major bovid radiation starting some 2.8 Ma and spurred by the onset of the modern Ice Ages, a climatic episode that also triggered faunal pulses in other African mammalian groups including our own lineage [39], [9].

Our results also reveal that cervids show higher specialization in tropical deciduous woodlands (II) and sclerophyllous woodlands (IV), which are forested biomes with a severe hydric seasonality. As proposed by Morales *et al.* [40], the ancestral acquisition of antlers in primitive deer could be the result of a “bone sink” system to maintain homeostasis during severe constraints of resources as a response to the onset of higher seasonality and stepper latitudinal gradient during the late early Miocene [2], [41], [42]. Additionally, recent studies on dental wear depict an ancestral facultative mixed feeding in basal cervids as early as the early Miocene [43]. Such facultative flexible diet may increase the resistance when marked seasonality restricts the nourishing vegetal resources. In the light of these evidences and our own results, we suggest that deer lineages have presented, from early stages of their evolutionary history, significant ecophysiological adaptations that enable them to overcome the environmental demands of highly seasonal woodlands. This has allowed cervids to take advantage of these ecosystems in comparison with other taxa. The conjunction of this physiological adaptation to hydric seasonality with the cyclic fragmentation and associated vicariance in tropical deciduous woodlands (II) at the global scales, as commented above, and sclerophyllous woodlands (IV) around the Mediterranean Basin gave rise to the high proportion of specialist cervids in both biomes.

All these findings strongly support the resource-use hypothesis. While conceding biogeographic history and physiological constraints certain role modulating speciation across biomes, we have demonstrated that, at a global scale, more specialist ruminants are prone to speciate at higher rates. From an empirical perspective, this is the first time that differential speciation rates of specialist lineages have been directly tested in a phylogenetic framework. Testing Vrba's predictions in other mammalian clades (including carnivores and fossil taxa) will shed valuable light on the universality of our conclusions. Although some authors have proposed that high extinction rates may accompany high speciation rates [44], [45], directly testing for the effect of biomic specialization on the extinction rates remains a problematic task. In our opinion, a correct assessment of trait-dependent extinction rates lies in the addition of fossil taxa. This, in turn, would involve the construction of a species level phylogeny of both living and fossil taxa and to gather a complete database of the traits under study. Nonetheless, despite their vast fossil record, we are still far from achieving such a precise knowledge on key ecological traits (e.g. biomic distribution, ecological niche, diet, etc.) of the known fossil ruminants or other fossil mammalian clades. A huge effort is being done in this field, though. And we think that the study of macroevolution and ecology will greatly profit from an inference framework where fossils, modern species and phylogenetic data can all be integrated.

### Conclusions

Our results agree with the predictions of the resource-use hypothesis proposed by Vrba. We found high frequency of species restricted to a single biome (BSI = 1) as a consequence of high speciation rates in stenobiomic lineages and higher specialization of species inhabiting biomes that underwent a high degree of fragmentation and contraction during climatic cycles: tropical rain forest (I), subtropical desert (III), steppe (VII) and tundra (IX). We also found significantly higher specialization among the species inhabiting tropical deciduous woodland (II), which also underwent expansions and retractions of its area during climatic cycles, providing a suitable scenario for speciation. Our findings strongly suggest that the difference in the biogeographic history, resource requirements and adaptations of bovids and cervids have marked their disparate specialization across biomes.

## Materials and Methods

### Data

For each species, we computed the biomic specialization index (BSI) developed by Hernández Fernández and Vrba [14], which is the number of biomes that it inhabits. We follow the biome classification of Walter [46], summarized in Table 8. The starting point of our data set consists of the complete geographical distributions of all 197 species of the suborder Ruminantia, encompassing living species and those that became extinct in the last two centuries [47], [27], [48]–[52], [29], [53], [54]. Distribution areas due to introduction by humans in historic times were omitted. For taxonomic consistency, we have followed the species-level taxonomy proposed by Wilson and Reeder [55]. The number of climatic zones inhabited by a species was assessed by the relative size of its geographic range in relation to the distribution of the different biomes and climatic dominions [56]. If 15% or more of the geographical range of a species is situated within a climate zone, the species was recorded as present in that climate zone. Since some climatic dominions are small enough to comprise less than 15% of the total distribution ranges of species with large range sizes, a species was also recorded as present in a specific climate zone if it inhabits 50% or more of one climatic dominion. We also considered those species inhabiting mountainous ranges as adapted to the biomes represented by analogous habitat series of altitudinal gradients, since these habitats present very similar vegetation physiognomy, ecological pressures and fragmentation dynamics during climatic fluctuations. We define stenobiomic species (biomic specialists) as those species inhabiting only one biome (BSI = 1). In turn, eurybiomic species (biomic generalists) are usually defined as those that occupy two or more biomes. Since species inhabiting five or more biomes must face very assorted environment conditions in terms of temperature and rainfall, Hernández Fernández and Vrba [14] proposed that this latter category may be subdivided in two other groups: “semi-eurybiomic species” including species with 1<BSI<5, and “extreme eurybiomic species” with species with BSI≥5.

**Table 8:** Biome typology used in this work (modified from Walter [46]).

Biome	
I	Evergreen tropical rainforest
II	Tropical deciduous woodland
II/III	Savannah
III	Sub-tropical desert
IV	Sclerophyllous woodland and shrubland
V	Temperate evergreen forest
VI	Broad-leaf deciduous forest
VII	Steppe/cold desert
VIII	Boreal coniferous forest (taiga)
IX	Tundra

### Analyses

#### i. Monte Carlo Simulations

We tested the resource-use hypothesis, which predicts uneven distributions of biomic specialists across clades and biomes following the mentioned predictions (see Introduction), against null models where biomic specialists are randomly distributed. We did so by comparing biomic presence–absence matrix with those generated by Monte Carlo randomizations of the observed data [14], [19]. Importantly, in each biome particular ecological features have an effect on species richness in such a way that there is no reason to consider that all the biomes must have the same number of species. Therefore, we conducted a simulation that places species in biomes randomly while constraining the observed species richness in each biome [14]. BSI null distributions and frequencies were obtained from 10000 random draws. The probability (*p-value*) that a BSI value could obtain by chance a percentage greater than the observed is obtained from the proportion of null values that are above the observed percentage; alternatively, the fraction of null values below the observed is the probability of obtaining a percentage of species less than the observed value. We used R [57] to perform all the analyses.

#### ii. BSI-dependent Speciation Models

**Figure 5:**
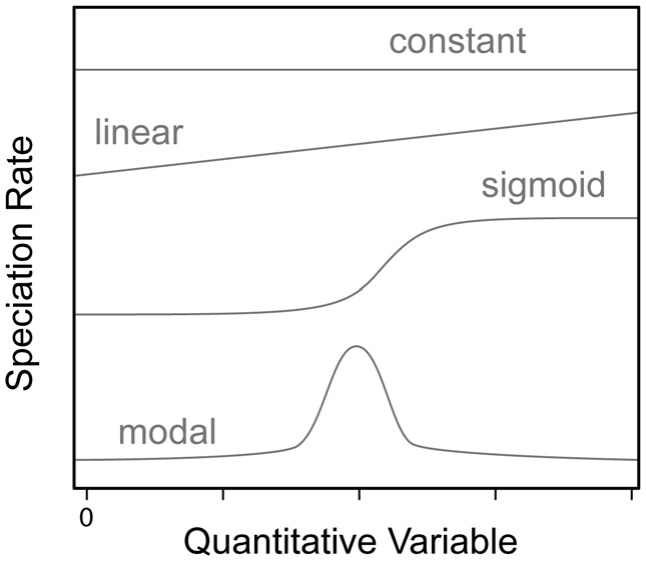
Quantitative-trait-dependent speciation models (QuaSSE). Representation of the maximum likelihood speciation rate models compared with QuaSSE, where the rate of speciation changes as a function of the variable under study, BSI in our case.

New likelihood-based methods provide a novel approach for identifying trait-dependent speciation rates [58], [59]. We explored the influence of biomic specialization on the speciation rates of the ruminants by applying quantitative-state speciation and extinction models (QuaSSE; [59]), where BSI was treated as a quantitative variable, on the phylogenetic tree of the group (see Fig. 1; [60]). Although extinction rates recovered from extant species phylogenies are largely underestimated [59], (but see Discussion), methods as the one used here provide accurate estimations of speciation rates [59]. Using QuaSSE we tested whether speciation rates have been higher in lineages showing BSI = 1 by comparing different models where BSI affects the speciation rate following constant, linear, sigmoidal and modal functions (Fig. 5) [59]. QuaSSE also allows identifying a directional or deterministic component in character evolution through the history of the group that may increase the fit of the model. Since QuaSSe requires the tree to be completely resolved, the polytomies were broken and a distribution of 100 fully dichotomous trees was produced following Kuhn et al. [62]. The models were run over each of the 100 trees and compared using their AIC scores [63], which measures the goodness of the fit of a statistical model while penalizing the number of parameters (the complexity) of the model. The best model gets the lower AIC score, and the fit of a model is significantly better than others when the difference between their AIC scores is greater than two units. The estimate of parameters was assessed from the 100 trees distributions. We performed QuaSSE in the statistical software R [57] as implemented in the diversitree library [64].
